# Capturing Biology in Flight

**DOI:** 10.1007/s13361-018-2084-x

**Published:** 2018-12-06

**Authors:** Carol V. Robinson

**Affiliations:** Physical & Theoretical Chemistry Laboratory, University of Oxford, South Parks Road, Oxford, OX1 3QZ UK

From the fundamental design of instrumentation to applications of ion mobility and the study of protein folding and interactions, I am honoured to read the articles in this special issue. Not only does the breadth of the science strike me but also the tremendous progress that has been made over the last few decades. As an introduction to these articles, I trace briefly the history of the mass spectrometry of macromolecular complexes. In so doing, I acknowledge the outstanding efforts of my colleagues worldwide, as well as former group members, in defining and establishing the field of gas phase structural biology and making it the highly respected research area it is today.

Back in the 1990s, it was considered counterintuitive to imagine that macromolecular assemblies could be preserved in the mass spectrometer. I still remember my initial excitement, when conducting experiments myself, upon finding conditions whereby it was possible to maintain and ‘fly’ intact non-covalent complexes of transthyretin and retinol-binding protein extracted from chicken plasma [1] and the *E. coli* GroEL _14-mer_ [2]. Today GroEL has become a benchmark for testing resolution and for tuning the properties of a mass spectrometer, a phenomenon which would not have been conceivable previously.

The Heck group was also working in this area, making major advances in studying macromolecular interactions including those between GroEL and the heptameric co-chaperonins (GroES and gp31) [3]. Both co-chaperonins assist in the folding of non-native polypeptide chains. Here, by demonstrating distinctive breakdown curves, and collision-induced unfolding patterns, the Heck group is able to conclude that GroES heptamers are strikingly more stable than the gp31 heptamers.

During these early days of studying protein interactions in vacuum, I became aware of the pioneering work of Joe Loo and his studies of ligand binding [4]. Previously, Joe’s group described first steps towards the tantalising prospect of defining non-covalent ligand-binding sites within proteins via top-down approaches [5]. Here, using electron capture dissociation (ECD), they are able to obtain sequence information for an intact protein, map phosphorylation sites, and pinpoint the sites of inhibitor binding.

Inducing fragmentation or unfolding via collisions is relatively straightforward to achieve albeit sometimes difficult to interpret. New ways of activating molecules in more controlled ways are coming to the fore. Combining ultra violet photo dissociation (UV-PD) with ion mobility (IM), Perdita Barran’s group shows here how they can control the initial unfolding steps of three proteins. Compact structures are populated during controlled UV-PD resulting in less fragmentation than the more extended structures that emerge following progressive in-source activation. By maximising selective cleavage at aspartic acid and proline residues, Scott McLuckey and his group use a sequence of proton transfer reactions to control charge and concentrate the precursor ions into one peak within a predefined *m*/*z* range, endowing enhanced selectivity and sensitivity to the fragmentation process.

Early methods of probing folded structure relied heavily on applications of hydrogen deuterium exchange—an approach that it is still widely employed due to its exquisite sensitivity. In this issue, three former group members (Justin Benesch, Argyris Politis and Antoni Borysik) advance both the practice and understanding of this methodology.

A longstanding interest of the Benesch group has been to understand the asymmetric charge partitioning that takes place during the collision-induced dissociation (CID) of protein complexes [6]. Joining forces with the research group of Kasper Rand, they present an interesting study in which they use gas phase H/D exchange not only to probe subunit interfaces but also to monitor their dissociation. They show that the deuterium content of the gas phase dissociated monomer can be used as a proxy for the deuterium uptake of the intact complex since only limited intermolecular H/D migration takes place during CID. Unexpectedly, this is also the case when very asymmetric charge partitioning occurs during dissociation.

Using HDX-MS to discriminate between native and non-native protein conformations, the Borysik group demonstrate remarkable correspondence between experimental and simulated data. A limitation that they highlight however is in the characterisation of homo-protein complexes. Knowledge of the native interface is required, from high-resolution structural information. By contrast, this is not a requirement for hetero-complexes wherein the degree of peptide sampling in the native interface can be inferred directly from differences in associated and free proteins. A need for more advanced algorithms for calculating protection factors is proposed. The work of Jürgen Claesen and Argyris Politis, in taking into account protein motions, and proposing a new method for calculating protection factors, is an important advance in this regard.

The strength of many mass spectrometry approaches is enhanced considerably when paired with other techniques. Two influential pioneers in applications of IM, David Clemmer and Mike Bowers, demonstrate this admirably by coupling IM with circular dichroism (CD) and atomic force microscopy (AFM) respectively. Research from the Clemmer group highlights the ability to monitor cis-trans isomerisation of polyproline examining interconversion of no less than eight different conformers. Meanwhile, combining IM with AFM, the Bower’s group tests the efficacy of a small molecule to inhibit the formation of the toxic Aβ42 dodecamer. The small molecule was found to prevent further aggregation of Aβ42, to destabilise preformed fibrils, and to reverse Aβ42 aggregation.

Continuing the theme of IM, I am grateful to my former group member Brandon Ruotolo. Following excellent graduate training from David Russell, Brandon convinced me that we should apply IM-MS to protein complexes. Following months of modelling, and coinciding with the construction of a commercial instrument, Brandon demonstrated that the overall topology of a ring-shaped protein complex could be retained in the gas phase [7]. He went on to study collision-induced unfolding, first in my group, where he supervised my daughter leading to my one and only mother and daughter paper [8]; later in his independent group, he continues to pioneer its application to biopharma products. Here, his researchers employ collision-induced unfolding to identify different peptide-binding sites within a kinase-inducible domain of a transcriptional coactivator.

During early applications of IM to protein assemblies, we considered at length how best to model protein assemblies and to assign the mass spectra of heterogeneous systems. Again, I am indebted to former group members (Tara Pukala, Matteo Degiacomi and Michael Marty) who transformed our ability to model complexes and to assign spectra respectively. Tara Pukala showed that by decomposing complexes into subassemblies, it was possible to construct models of interacting subunits and to assemble complexes of unknown topology [9]. Herein, she takes on a new direction in her IM and modelling of DNA and RNA triplexes.

Central to all modelling strategies is the representation of protein subunits. Matteo Degiacomi proposes a new expression to describe the overlap between two spheres for coarse-grained modelling. Also, in this issue, Michael Marty builds on his powerful deconvolution software UniDec [10] with the introduction of MetaUniDec. He illustrates the power of this software through application to automated collision voltage ramps applied to a small bacterial heme protein and large lipoprotein nanodiscs. A discrete loss of heme is observed for the protein; by contrast, nanodiscs show a continuous loss of lipids and charge as result of increasing collisional activation.

While fundamental studies pave the way for everything we do, I continue to be excited by the novel insight and biological knowledge that can be attained from the study of intransigent systems using MS. The interplay between phosphorylation status and interactions is beautifully demonstrated in the research of Claire Eyres. For example, specific phosphorylation events are shown to regulate the functions of homodimers and DNA binding.

Combining cross-linking with MS provides structural details for interaction partners. The ability to carry out cross-linking in a reproducible and informative way remains challenging largely due to the paucity of cross linkers and robust software for interpretation [11]. Both these limitations are disappearing and in this issue, Andrea Sinz exploits the power of new cleavable cross-linkers introducing the first CID-MS/MS-cleavable, photo-thiol-reactive cross-linker (*1,3-*diallylurea).

Continuing with former group members, I am delighted to see the contributed article from Carla Schmidt and her research group. Carla first introduced me to the powers of cross-linking showing how differential cross-linking could be used to distinguish different populations of complexes—with or without phosphorylation for example [12]. Here, she studies the oligomerisation properties of three Synaptobrevin-2 variants either in the absence of interaction partners or when incorporated into the lipid bilayer of liposomes. Combining mass spectrometry of the oligomers with chemical cross-linking, they confirm the presence of oligomers in solution while membrane-bound Synaptobrevin-2 is essentially monomeric.

It is, however, integral membrane proteins and their properties and structures in the gas phase that have always fascinated me. My adventures in this challenging area began over a decade ago first with Leopold Ilag [13] and later when Nelson Barrera entered my laboratory. Using detergent micelles to protect membrane proteins and release them into the gas phase, we found that we could maintain interactions between subunits [14]. Designing detergents that are suitable for protein MS represents an on-going challenge. Desirable characteristics for a detergent include charge-reducing properties and low gas phase stabilities of complexes formed with proteins. Research presented here from Kevin Pagel, also a former group member who explored the relationship between charge manipulation and dissociation pathways [15], exploits the gas phase properties of oligoglycerol detergents fine-tuning their molecular structure to produce charge reduction and compromised stability of the resulting protein detergent complex.

Lipids were discovered in these membrane protein complexes in detergent micelles and through my interactions with Min Zhou and Nina Morgner, we uncovered roles for lipids in rotary ATPases [16]. Research in the Morgner group reported here compares properties of membrane proteins generated through nanoelectrospray and LILBID and highlights the complementarity of both approaches.

Through my interactions with Art Laganowsky and others, we learnt much about the modulation of protein structures through specific lipid interactions [17]. The mass and mobility resolution we could achieve at that time was limiting however. Developments in Orbitrap mass spectrometers [18, 19] unravelled the heterogeneity of different lipid families and thus combining high-resolution IM-MS with an Orbitrap mass analyser has become a long-held dream. Moving one step closer to realising this goal Art Laganowsky and David Russell describe here a novel reverse-entry ion source (REIS) coupled to an Orbitrap with extended mass range. Ubiquitin (8.5 kDa) and lipid binding to the membrane protein ammonia transport channel (AmtB, 126 kDa) demonstrate the performance of the REIS instrument and pave the way for a future whereby high-resolution mass measurement and IM will *finally* be possible.

I am proud that many of my former group members have gone on to forge careers at least in part influenced by their time spent in my laboratory. It is equally rewarding to see former group members embark on entirely new directions. Leopold Ilag while in my group looked at early applications of MS to membrane proteins [13]. Here, in a complete change of direction, he reports new methods to look at the proteinaceous content of size-resolved aerosols collected in the Arctic.

Whether it is proteins from Arctic aerosols, the cell or its membrane, or the design of a new instrument, over my research career, I have witnessed great progress in the mass spectrometry of protein interactions, events which turned early scepticism to international acceptance. Moreover, researchers in this field continue to uncover new insights, to inspire novel research and to prompt new hypotheses. It remains for me to thank colleagues, friends, and current and former group members who have, over the years, played such an important part in inspiring my own work and have been instrumental in ‘capturing biology in flight’.
